# A20 promotes colorectal cancer immune evasion by upregulating STC1 expression to block “eat-me” signal

**DOI:** 10.1038/s41392-023-01545-x

**Published:** 2023-08-23

**Authors:** Min Luo, Xueping Wang, Shaocong Wu, Chuan Yang, Qiao Su, Lamei Huang, Kai Fu, Sainan An, Fachao Xie, Kenneth Kin Wah To, Fang Wang, Liwu Fu

**Affiliations:** 1 State Key Laboratory of Oncology in South China, Collaborative Innovation Center for Cancer Medicine, Guangdong Esophageal Cancer Institute, Sun Yat-sen University Cancer Center, Guangzhou, 510060 P.R. China; 2 Laboratory Animal Centre, The First Affiliated Hospital of Sun Yat-sen University, Guangzhou, 510080 P.R. China; 3 School of Pharmacy, The Chinese University of Hong Kong, Hong Kong, China

**Keywords:** Cancer microenvironment, Gastrointestinal cancer, Tumour immunology

## Abstract

Immune checkpoint inhibitors (ICIs) have induced durable clinical responses in a subset of patients with colorectal cancer (CRC). However, the dis-satisfactory response rate and the lack of appropriate biomarkers for selecting suitable patients to be treated with ICIs pose a major challenge to current immunotherapies. Inflammation-related molecule A20 is closely related to cancer immune response, but the effect of A20 on “eat-me” signal and immunotherapy efficacy remains elusive. We found that A20 downregulation prominently improved the antitumor immune response and the efficacy of PD-1 inhibitor in CRC in vitro and in vivo. Higher A20 expression was associated with less infiltration of immune cells including CD3 (+), CD8 (+) T cells and macrophages in CRC tissues and also poorer prognosis. Gain- and loss-A20 functional studies proved that A20 could decrease the “eat-me” signal calreticulin (CRT) protein on cell membrane translocation via upregulating stanniocalcin 1 (STC1), binding to CRT and detaining in mitochondria. Mechanistically, A20 inhibited GSK3β phosphorylating STC1 at Thr86 to slow down the degradation of STC1 protein. Our findings reveal a new crosstalk between inflammatory molecule A20 and “eat-me” signal in CRC, which may represent a novel predictive biomarker for selecting CRC patients most likely to benefit from ICI therapy.

## Graphical abstract

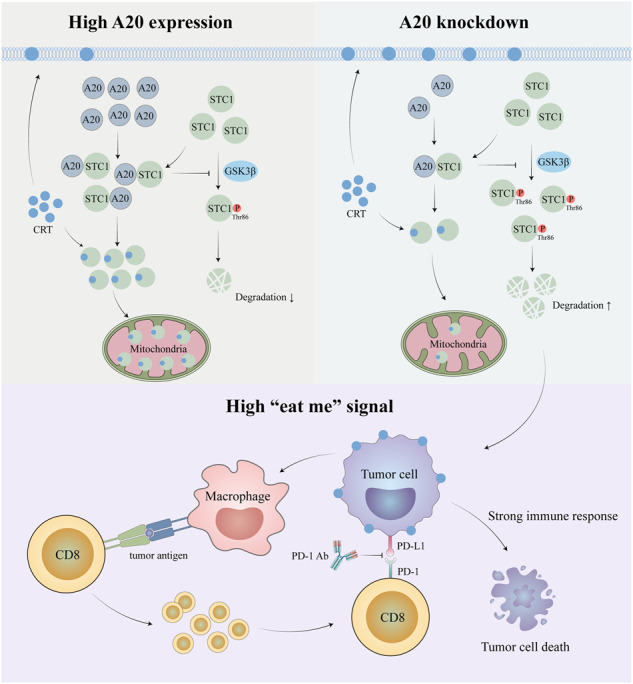

## Introduction

Colorectal cancer (CRC) is one of the most common malignant tumors. The 5-year survival rate was about 90% in patients with localized primary disease, 70.4% in those with lymph node or peripheral metastasis, and 12.5% in those with distant metastasis.^1^ The immune checkpoint inhibitors (ICIs) have been demonstrated a promising therapy in many malignancies, including microsatellite instability (MSI)-high colorectal cancers (CRCs).^2,3^ ICI works by overcoming or alleviating tumor-induced immunosuppression, thereby enhancing the immune response against cancer cells and thereby eliminating tumors. It is known that the abundance of inflammatory cells infiltrating in the tumor microenvironment (TME) is strongly associated with the efficacy of ICIs.^4^ There are four sub-types of TME according to the tumor mutation burden (TMB) and the expression levels of T-cell inflammatory genes.^5^ CRCs are usually divided into two types, MSI-high and microsatellite-stable (MSS). Higher somatic TMB is more likely to express immunogenic neo-antigens,^6^ allowing them to be responsive to ICIs.^7,8^ In contrast, low TMB and low inflammatory gene signature often manifest as a phenotype of immune desert or immune cell rejection, which is associated with inefficient or non-existent antigen presentation and thus an inferior adaptive immune response.^9,10^ Therefore, patients with low TMB and low inflammatory gene markers usually exhibit poor clinical outcomes when receiving immunotherapies.

Despite of the extended survival, many patients have innate or acquired resistance to ICIs. The low response rate and the lack of appropriate biomarker for selecting the right patients for ICI treatment are severely limiting the efficacy of ICIs in clinic. The initiation of adaptive immune response to tumor antigens requires the uptake of antigen presenting cells (APCs) and its presentation to naive T cells.^11^ To this end, infiltration of APCs into tumor tissues is defective in tumors with low TMB and low inflammatory gene signature. Thus, novel therapies aimed at overcoming this limitation and further supporting T cell function in tumor tissue may be of greatest benefit in patients with tumors of this type.

Phagocytosis is important in surveillance against cancer.^12^ It is regulated by several molecular “eat me” signals on cell surface of cancer cells, which allow recognition of extracellular cargos and subsequently activating phagocytic receptors and other downstream signaling cascades.^13^ Calreticulin (CRT) is a 46 kda soluble protein that is primarily localized to the endoplasmic reticulum (ER) and is considered an important“eat me” signal.^14^ In pro-apoptotic cancer cells, CRT shifted onto the cell membrane as an “eat me” signal, which is recognized by APCs (such as DCs and macrophages) to activate specific T cells.^15^ APCs prime and activate specific T cells and are critical for defining the success of ICIs therapy. This process is dependent on whether APCs could efficiently capture antigens from pro-apoptotic tumor cells via phagocytosis, present sufficient antigens to T cells, and activate T cells.^16^ Therefore, the membrane translocation of CRT is a key step in the successful treatment of ICI. Stanniocalcin-1(STC1) is a hormone-like glycoprotein that has been shown to regulate calcium and phosphorus homeostasis.^17^ Tumorous STC1 could interact with CRT to trap CRT in mitochondria area, and then reduces membrane CRT.^18^ Consequently, membranous CRT-mediated phagocytosis by APCs is attenuated, resulting in impaired APC antigen presentation and T cell activation.^18^ It has been postulated that blocking the “eat me” signals or their receptors could impair APCs-mediated phagocytosis and induce therapeutic resistance to ICIs.^18^

Inflammation is closely associated with the development of many cancers, including colorectal cancer, and plays a key role in regulating cancer immune responses.^19^ A20, tumor necrosis factor inducible protein 3(TNFAIP3), is a potent anti-inflammatory enzyme can weaken the inflammatory signals mediated by cytokines and pathogens.^20^ A20 consists of an N-terminal ovarian tumor domain (OTU) with deubiquitinase activity and seven C-terminal cys2-cys2 zinc finger domain (znfs) with a total of 790 amino acid residues.^21,22^ The synergistic activity of these two ubiquitin-editing domains mediates the negative regulatory role of A20 in NF-κB signaling.^23^ Thus, polymorphisms or genetic defects of A20 gene may contribute to the initiation and progression of multiple autoimmune diseases through activation of pro-inflammatory NF-κB signaling.^24^ Our study bring to light a novel crosstalk between inflammatory molecules A20 and “eat-me” signal in CRC, providing a new biomarker for selecting appropriate patients for ICIs and a potential ICI combination therapeutic strategy in CRC. In our study, the upregulation of A20 in cancer cells promoted the growth of CRC via reducing the cell surface expression of CRT protein and facilitating tumor immune evasion in a STC1-CRT–dependent manner. High expression of A20 was associated with poor response to PD-1 inhibitor therapy and negatively with survival rate of patients with CRC. Gene overexpression and knockout experiments demonstrated that A20 promoted tumor immune evasion and induced resistance to ICIs in vitro and in vivo. Our results suggest that A20 acts as an intracellular “eat-me” signal blocker. Targeting A20 represents a novel approach to overcome ICI resistance in cancer therapy.

## Results

### High A20 expression is associated with poor immune cells infiltration in CRC

**Fig. 1:**
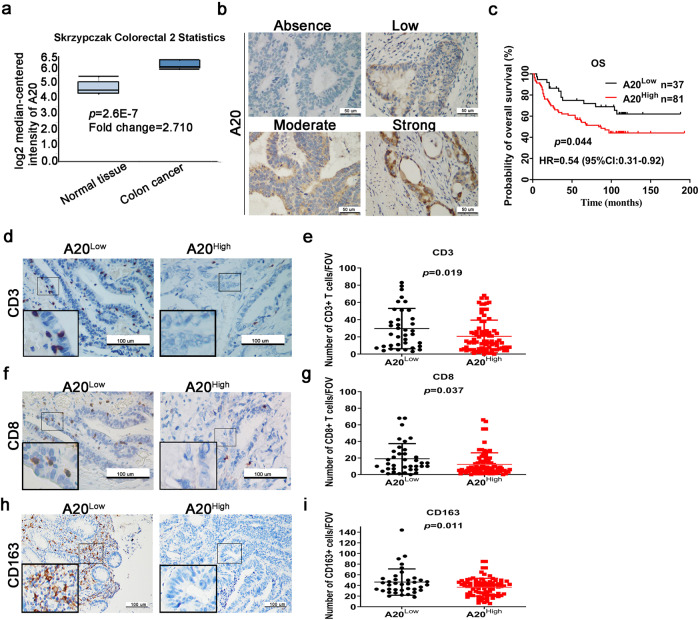
A20 expression is associated with poor immune cell infiltration. **a** A20 expression in normal tissue and colon tumor analyzed by Oncomine database. **b** The representative images of different intensity of tumorous A20 expression from human CRC tissue samples with A20 immunohistochemical staining (×200). The four representative pictures are not from CRC patient at different stage, just with different A20 intensity. **c** The survival analysis of 118 CRC patients. H-score ≤ 6, low; >6, high. **d**–**i** The representative pictures showing the extent of immune cell infiltration in tumor microenvironment in CRC specimens. The numbers of immune cells were averaged by five different fields of views and the data were presented as mean ± SD. *p < 0.05; **p < 0.01; ***p < 0.001; n.s. not significant

Using the cancer microarray database and comprehensive data-mining platform ONCOMINE (www.oncomine.org), the expression of A20 in CRC tissues was significantly higher than that in normal tissues (Fig. 1a). The infiltration of immune cells in tumors was linked to the efficacy of ICI treatment.^4^ To evaluate whether A20 expression was associated with immune cells infiltration and prognosis of CRC patients, immunostaining using specific antibodies against tumorous A20 and a few immune cell markers in immune cells was conducted in 118 cases of CRC tumor specimens (Fig. 1b).

According to the survival analysis CRC patients with high A20 expression (H-score ≥6) exhibited poorer overall survival than those with low A20 expression (H-score<6) (Fig. 1c). The abundance of CD3(+) and CD8(+) infiltrating lymphocyte and macrophages (CD163(+)) in tumor tissues were significantly decreased in A20-overexpressing tumor tissues (Fig. 1d–i). Collectively, tumoral expression of A20 was found to be inversely correlated with immune cell infiltration and therapeutic effect of ICI treatment.

### A20 inhibited anti-tumor immunotherapeutic response in CRC in vitro

**Fig. 2:**
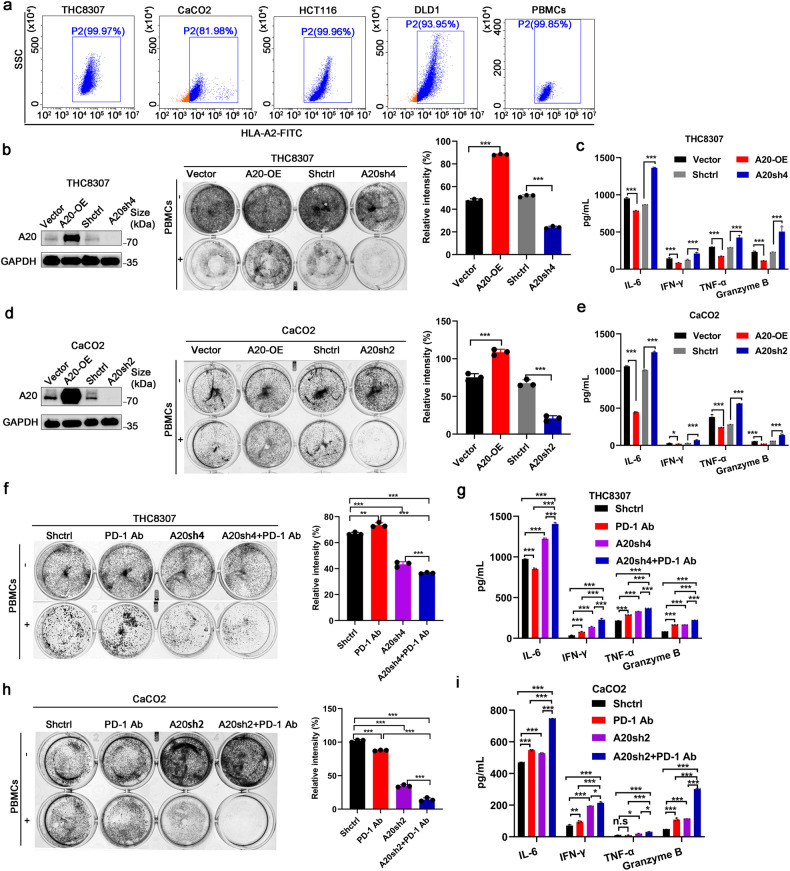
A20 inhibited anti-tumor immune response in vitro. **a** The flow cytometry analysis of HLA-A2. **b** The lymphocytoxicity effect of PBMCs on THC8307 cells when A20 expression was manipulated by overexpression (OE) or short hairpin silencing (sh). **c** The ELISA analysis of T cell activation-related cytokines from the co-culture medium of THC8307 cells and PBMCs, *n* = 3. **d**, **e** The effect of A20 expression on lymphocytoxicity and cytokine release from the co-culture medium of CaCO2 cells and PBMCs, *n* = 3. **f**–**i** The effect of PD-1 inhibitor on lymphocytoxicity and cytokine release from the co-culture medium of A20-knockdown CRC cells and PBMCs, *n* = 3. All experiments were performed in triplicate. Data was represented as the mean ± SD. *p < 0.05; **p < 0.01; ***p < 0.001; n.s not significant

Forced expression or genetic silencing of A20 was conducted in a few CRC cell lines to investigate the role of A20 expression on anticancer immunotherapeutic response. The A20 expression level in different CRC cell lines was detected (supplementary Fig. 1a). A20 expression level did not alter the cell proliferation in CRC cells in vitro (supplementary Fig. 1b–f). However, significantly more tumor cell deaths and less secretions of cytokines (IL-6, IFN-γ, granzyme B and TNF-α) from the medium were observed in CRC cells with A20 overexpression than that in control cells and PBMCs co-culture system with matched HLA-A2 (Fig. 2a–e and Supplementary Fig. 1g–k). These results suggest that A20 overexpression inhibited the lymphocytoxicity of PBMCs and the function of immune cells.

Furthermore, to further investigate the effect of A20 on anticancer effect of PBMCs in the presence or absence of ICIs, the human PD-1 antibody was added to the co-culture system of CRC cells and PBMCs. A significant enhancement of lymphocytoxicity of PBMCs and its secretion of cytokines (IL-6, IFN-γ, granzyme B, and TNF-α) were observed in the presence of PD-1 inhibitor than the control in co-culture system of A20-knockdown CRC cells and PBMCs (Fig. 2f–i and Supplementary Fig. 1l–o). These findings suggest that the deletion of A20 induces a stronger antitumor immune response in CRC cells in vitro.

### A20 inhibited anti-tumor immunotherapeutic response in CRC in vivo

**Fig. 3:**
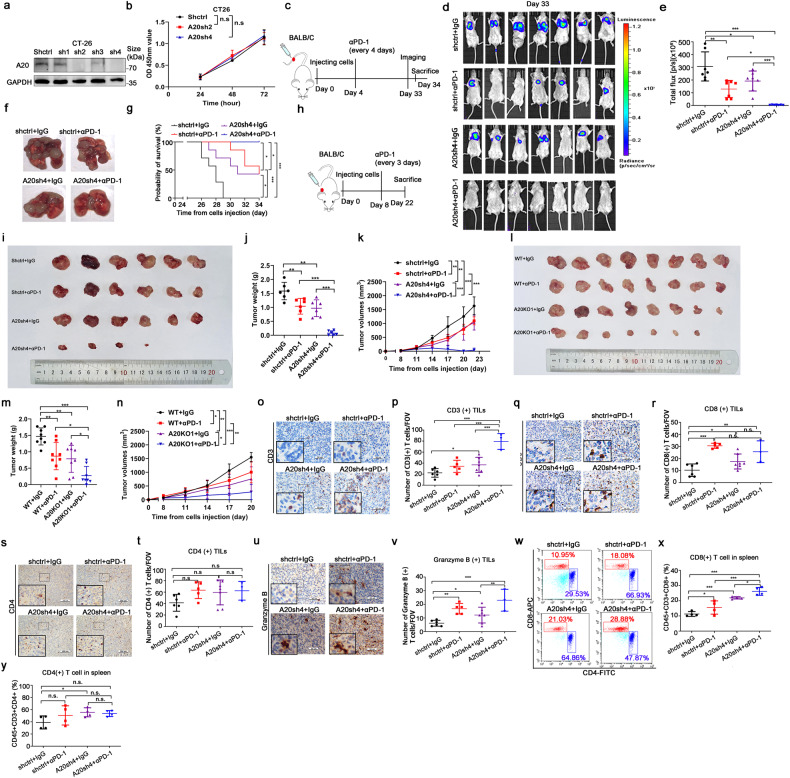
A20 inhibited antitumor immune response in vivo. **a** The expression of A20 in mice CT26-luc-GFP cells. **b** The cell proliferation of CT26-luc-GFP cells detected by CCK8 kit, *n* = 4. **c** The experimental scheme of the animal study. **d** The in vivo images of mice tumors with different treatments were detected by the IVIS bioluminescence imaging system, *n* = 7. **e** Statistical analysis of total flux from the IVIS bioluminescence images. **f** The representative images of the metastatic nodes in lung from BALB/C mice. **g** The survival curve of mice, *n* = 7. **h** The experimental scheme of the animal study. **i** The images of tumors excised from BALB/C mice at the end of experiments, *n* = 6. **j** Tumor weights of the four treatment groups, *n* = 6. **k** Tumor growth curves of the four treatment groups, *n* = 6. **l** The images of tumors excised from BALB/C mice at the end of experiments, *n* = 8. **m** Tumor weights of the four treatment groups, *n* = 8. **n** Tumor growth curves of the four treatment groups, *n* = 8. **o**–**v** The infiltration of CD3 (+) (X400), CD8 (+) (X400), CD4 (+) (X200), and Granzyme B(+) (X400) T cells detected by immunohistochemical staining. *p*-value was calculated by one-way ANOVA analysis. Shctrl+IgG, *n* = 7, Shctrl+αPD-1, *n* = 5, A20sh4+IgG, *n* = 7, A20sh4+αPD-1, *n* = 3. **w**–**y** Flow cytometry analysis of CD8 (+) and CD4 ( + ) T cells of mice spleens (*n* = 4 per group). Data was represented as the mean ± SD. *p < 0.05; **p < 0.01; ***p < 0.001; n.s. not significant

To explore whether A20 can attenuate the antitumor response of anti-PD-1 immunotherapy in vivo, we established a metastatic mouse model bearing CRC cells. The cell proliferation of CT-26 cells was not affected by A20 silence in vitro (Fig. 3a, b and supplementary Fig. 1f). Neither did cell migration or invasion potential was altered by A20 downregulation (supplementary Fig. 2a–e). The experimental scheme is depicted in Fig. 3c. Murine CT-26-luciferase-GFP cells infected with a specific shRNA targeting A20 or a mock shRNA control were injected into the tail veil of BALB/C mice. The mice were then treated with IgG control or anti-PD-1 antibody. Tumor growth in the lung was monitored by bioluminescence imaging. Tumor growth in lung was significantly inhibited and the number of tumor nodules in lung tissue was remarkably decreased by PD-1 antibody in mice bearing the A20-silent CT-26-luciferase-GFP cells, but only slightly inhibited in mice bearing the control CT-26-luciferase-GFP cells (Fig. 3d–f). Importantly, the overall survival was remarkably longer in mice bearing the A20-silent CT-26-luciferase-GFP cells than those bearing the control CT-26-luciferase-GFP cells treated with PD-1 antibody treatment (Fig. 3g). Likewise, the subcutaneous tumor growth was also significantly inhibited by PD-1 antibody in mice bearing the A20-silent CT-26 cells relative to mice bearing the control CT-26 cells (Fig. 3h–n). Thus, high expression of A20 was shown to attenuate the efficacy of PD-1 antibody in vivo.

The function of A20 on the TME and tumoral lymphocyte infiltration were also investigated. We found that CD3 (+) and CD8 (+) T cells except CD4 (+) T cells, were notably increased in tumor tissues from lungs upon genetic silencing of A20 (Fig. 3o–t). Importantly, the amount of granzyme B(+) immune cells was also significantly increased in the TME in mice bearing the A20-knockdown tumor (Fig. 3u, v). Furthermore, the percentages of CD8 (+) T cells rather than CD4 (+) T cells were also remarkably increased from spleens tissues in mice bearing A20-silent tumor (Fig. 3w–y). Collectively, these findings indicate that A20 suppressed the infiltration and activities of immune cells in tumor tissues.

### A20-mediated tumor immune evasion led to impaired phagocytosis function of macrophages

**Fig. 4:**
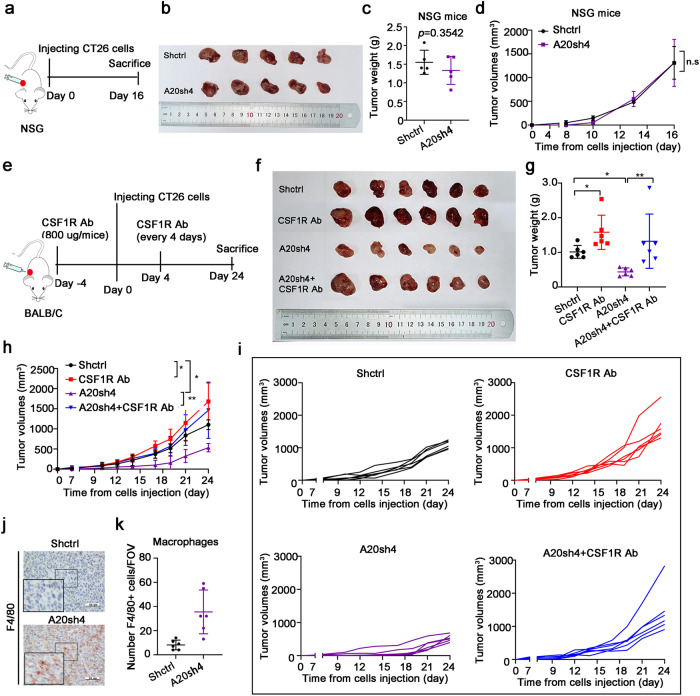
A20 impaired the phagocytosis function of macrophages to mediate tumor immune evasion. **a** The experimental scheme of the animal study. **b** The images of tumors excised from NSG mice at the end of experiments. **c** Tumor weights of the two treatment groups, *n* = 5. **d** Tumor growth curves of the two treatment groups, *n* = 5. **e** The experimental scheme of the animal experiment using BALB/C mice. **f** The images of tumors excised from BALB/C mice at the end of experiments. **g** Tumor weights of different groups, *n* = 6. **h**, **i** Tumor growth curves, *n* = 6. **j**, **k** The infiltration of macrophages (F4/80+) in mice tumor tissues (X400), *n* = 6

Macrophage-mediated immune-surveillance plays a critical role in cancer immunotherapy. Cancer cells presenting the “eat-me” signal on their cell surface are recognized and phagocytosed by macrophage.^12^ Thus, impairment of the phagocytosis function of macrophages is associated with resistance of ICI therapy.^18^ To investigate whether A20-mediated tumor immune evasion was related to phagocytosis function of macrophages, the effect of CSF1R antibody (an inhibitor to deplete macrophages) on tumor growth inhibition was studied in a mouse model bearing CT26 cells. The expression level of A20 did not alter CRC cell proliferation in vitro Supplementary Fig. 1a–f) nor did they alter tumor xenograft growth in NSG mice (Fig. 4a–d). Consistent with the immune surveillance function of macrophages, the tumor xenografts in NSG mice bearing CT26 cells were found to grow faster in presence of CSF1R antibody than that in absence of CSF1R antibody (Fig. 4e–i). Importantly, CSF1R antibody impaired the tumor growth inhibition by A20 downregulation in the mice bearing A20-silent CT26 cells (Fig. 4e–i). These finding suggests that the promotion of tumor growth by A20 was dependent on the inhibition of phagocytosis function of macrophages. Importantly, the infiltration of F4/80 (+) macrophages was notably increased in the tumor tissue from the mice bearing A20-silent CT26 cells (Fig. 4j, k). Taken together, the loss or downregulation of A20 promoted the anti-tumor immune response in TME.

### A20 downregulation promoted the formation of antitumor immune microenvironment via STC1/ CRT signaling pathway

**Fig. 5:**
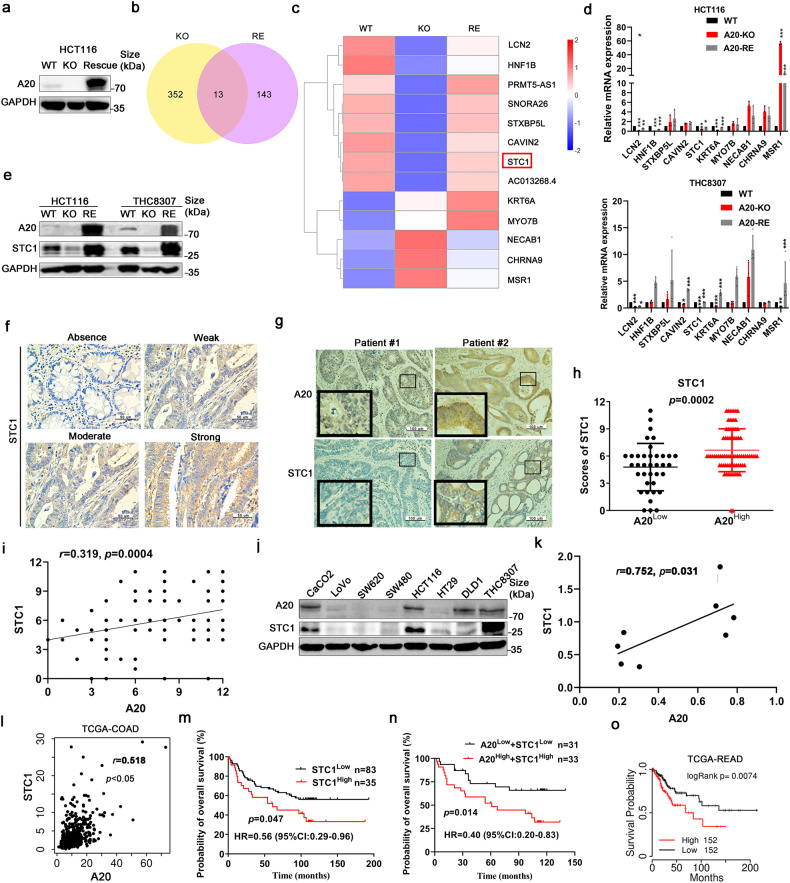
A20 regulated STC1 expression. **a** A20 expression level in A20-knockout (KO) and A20-KO-rescue (RE) cells. RNA-sequencing was conducted to investigate the gene profiles from HCT116 cells after manipulation of A20 expression (WT: wild type; KO: A20-knockout; RE: A20-rescue). **b** The Venn diagram of the genes altered by A20 with the *p*-value < 0.05. **c** Heat map of 13 genes (*n* = 3 samples of each group). Blue, downregulation; red, upregulation. **d** The expression of 10 genes were detected by q-PCR, *n* = 4. **e** The expression of STC detected by immunoblotting. **f** Representative immunohistochemical staining images of different intensity of STC1 expression in tumor specimens. **g** Representative images of STC1 and A20 staining in tumor specimens from CRC patients. **h** Statistical analysis of STC1 expression in CRC tissues with high or low A20 expression, *n* = 118. **i** Correlation analysis of STC1 and A20 expression in CRC tissues, *n* = 118. **j** Immunoblotting analysis of A20 and STC1 expression in CRC cell lines (*n* = 8). **k**, **l** Correlation analysis of A20 and STC1 expression in CRC cell lines and TCGA database. **m**, **n** Survival analysis of CRC patients (H-score≤6, low; >6, high). **o** Survival analysis of CRC patients in TCGA dataset

To understand the mechanism of macrophages infiltration in tumor tissues and the efficacy of anti-PD-1 therapy by A20 downregulation, A20-knockout (KO) and A20-KO-rescue (RE) models in HCT116 cells were established. The gene expression profiles in the KO and RE models were examined and analyzed by RNA-sequencing. In A20 KO cells, 352 genes were found to be altered with the log-fold >2 or < −2 compared to wild-type (WT) cells (Fig. 5a, b). Upon the rescue of A20 KO, 143 genes were significantly changed in A20-RE cells compared to A20-KO cells (Fig. 5b). It is noteworthy that 13 genes were consistently changed among these altered genes (Fig. 5b, c). And some of them play a role in innate immune response or calcium channel activity, like LCN2, KRT6A, NECAB1, CHRNA9, MSR1, and STC1. mRNA levels of a few differentially expressed genes from RNA-sequencing experiment were also confirmed by q-PCR, respectively, in HCT116 and THC8307 cells (Fig. 5d). Only LCN2, KRT6A, and STC1 were altered consistently with RNA-seq results. Interestingly, STC1 was reported to inhibit the antitumor immune response via decreasing the “eat-me” signal on tumor cell membranes.^18^ So we focused on STC1 and detected its protein expression by western blotting in HCT116 and THC8307 cells, respectively (Fig. 5e). The results showed that STC1 expression was decreased when A20 was knocked-out, but STC1 expression was increased when A20 was restored (Fig. 5d, e). Thus, STC1 expression was upregulated by A20 in CRC cells.

To further verify the association of A20 and STC1 expression in CRC tissues, immunohistochemical staining was carried out in tumor specimens from 118 CRC patients. The differential expression intensity of STC1 from different patients was illustrated by representative staining images in Fig. 5f. Importantly, tumor tissues with high A20 expression were also observed with high STC1 expression, and vice versa (Fig. 5g, h). A20 expression was positively correlated with STC1 expression in CRC specimens (Fig. 5i). Furthermore, A20 was positively correlated with STC1 expression in CRC cell lines (Fig. 5j, k) and TCGA datasets (Fig. 5l). Importantly, a much higher survival probability was observed in CRC patients with low STC1 expression than those with high STC1 expression (Fig. 5m, n). Similar findings were also obtained from TCGA datasets (Fig. 5o). These findings indicate that STC1 may play a key role in A20-mediated immune evasion in CRC.

**Fig. 6:**
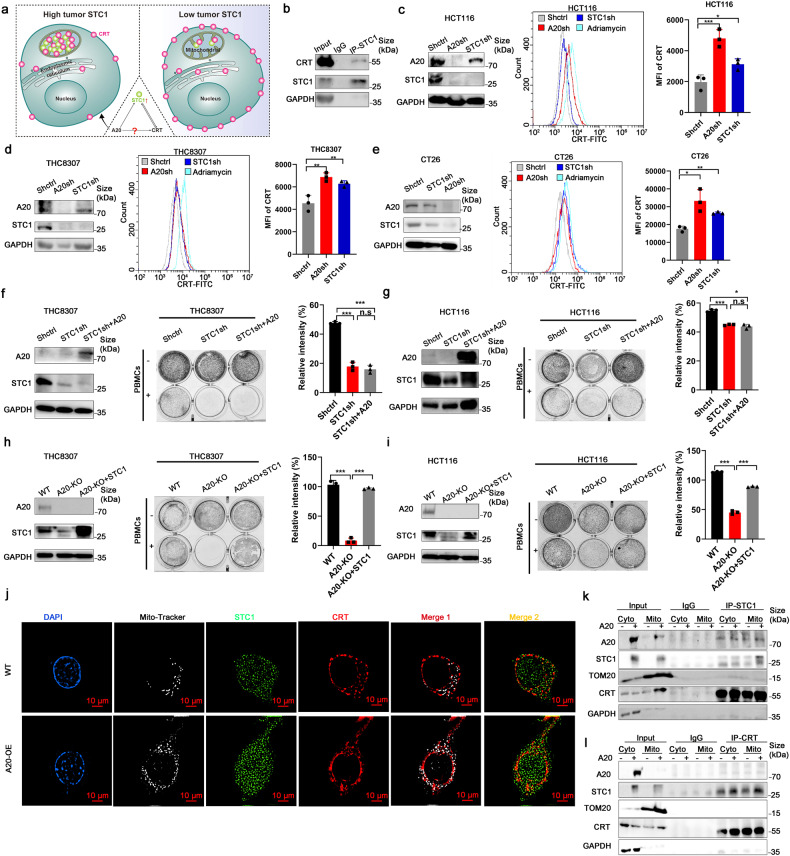
A20 inhibited antitumor immune response via STC1/ CRT signaling pathway. **a** Working model showing the crosstalk between A20 and CRT in CRC cells. **b** Co-immunoprecipitation analysis of STC1 and CRT. **c**–**e** The effect of STC1 or A20 downregulation on the translocation of CRT to cell membrane detected by flow cytometry. Adriamycin was used as a positive control. **f**, **g** The lymphocytoxity to THC8307 or HCT116 cells with A20 overexpression but STC1 downregulation, *n* = 3. **h**, **i** Co-culture experiment of PBMCs and A20-KO THC8307 or HCT116 cells rescued with STC1 overexpression, *n* = 3. Data was presented as the mean ± SD. *p < 0.05; **p < 0.01; ***p < 0.001; ****; *p* < 0.0001; n.s, not significant. **j** CaCO2 cells transfected with A20 overexpression vector were detected by immunofluorescence assay to show the interactions of CRT (red) and STC1 (green), co-localization with MitoTracker (white) (×1000). **k**, **l** The interactions of STC1 and CRT protein in cytoplasm and mitochondria of CaCO2 cells with A20 overexpression

It was reported that STC1 bound with CRT in mitochondria, thereby preventing the shift of CRT to the cell membrane, reducing the “eat-me” signals and protecting tumor cells from phagocytes by macrophages.^18^ We hypothesized that A20 induces STC1 overexpression and increases the retention of the CRT-STC1 complex in mitochondria, which subsequently inhibiting the shift of CRT to the cell membrane as an “eat-me” signal. The working model is depicted in Fig. 6a. The interaction of STC1 and CRT was demonstrated by co-immunoprecipitation (Fig. 6b). CRT expression on cell membrane was significantly increased when STC1 was silenced by shRNA in HCT116, THC8307 and CT26 cells (Fig. 6c–e and supplementary Fig. 3a). Moreover, the downregulation of A20 was also found to increase the shift of CRT protein to cell membrane (Fig. 6c–e). On the other hand, translocation of the “eat-me” signal CRT to cell membrane was significantly decreased in HCT116, THC8307 and CT26 cells upon the overexpression of STC1 or CRT (supplementary Fig. 3b–d).

To further elucidate whether STC1 is a key signaling molecule responsible for A20-mediated immune evasion, co-cultures of CRC cells (with or without STC1 silencing) and PBMCs were set up. In the co-culture system, viability of CRC cells were remarkably decreased in STC1-silent CRC cells relative to the control (Fig. 6f, g). Importantly, in STC1-silent CRCs, PBMC-mediated cytotoxicity was not attenuated by A20 overexpression (Fig. 6f, g). However, overexpression of STC1 could repress cell death of A20-silent CRC cells in co-culture with PBMCs (Fig. 6h, i). The effects of STC1 and A20 on the release of T cell activation-related cytokines (like TNF-α, granzyme B, IL-6 and IFN-γ) was also examined in the co-culture system. Significantly more TNF-α, granzyme B, IL-6, and IFN-γ was found to be released from PBMCs when they were in co-culture with STC1- or A20-silent CRC cells, compared with control CRC cells (supplementary Fig. 3e, f). However, the increase in cytokine release was abolished in the co-culture system by STC1 overexpression (supplementary Fig. 3g, h).

The effect of A20 on cell membrane translocation of CRT was investigated by confocal microscopy. In CRC cells overexpressing A20, an increased interaction of STC1 and CRT in the mitochondria was observed (Fig. 6j). More importantly, an increase in translocation of CRT to the cell membrane was evident when A20 was downregulated in CRC cells (Fig. 6c, d). To further elucidate whether more STC1-CRT complex was retained in the mitochondria after A20 overexpression, the two proteins were detected by Western blotting analysis in the mitochondrial and cytoplasmic fractions from the CRC cells. The results further confirmed that more STC1 and CRT were located in the mitochondria in A20-overexpressing CRC cells than in control cells (Fig. 6k, l). Furthermore, the binding of STC1 and CRT in the mitochondrial fraction was detected by co-immunoprecipitation in A20-overexpressing CRC cells than in control cells (Fig. 6k, l).

### A20 upregulated STC1 via inhibition of its protein degradation

**Fig. 7:**
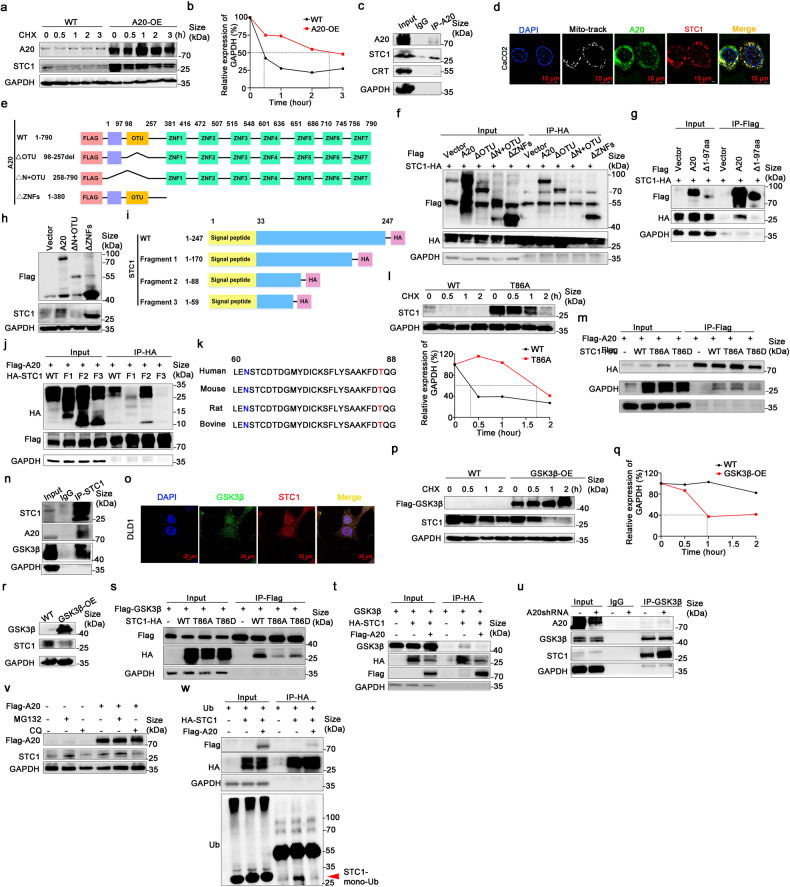
A20 upregulated STC1 by inhibiting STC1 degradation. **a**, **b** Degradation of STC1 protein over time in cells with or without A20 overexpression (OE). Cycloheximide was used at 20 μM. **c** The interaction between A20 and STC1 detected by Co-IP. **d** The co-localization of STC1 (green) and A20 (red) (×1000) detected by immunofluorescence assay. Blue dye, DAPI indicates the nucleus, MitoTracker (white). **e** Schematic diagram of A20 domains in the various constructs. **f**, **g** The interaction between STC1 and A20 with different domain-deletions. **h** The effect of A20 with different domain-deletions on STC1 expression. **i** Schematic diagram showing 4 STC1 expression vectors with different C-terminal deletions. **j** The interaction between A20 and STC1 (having different C-terminal deletions). **k** The amino acid sequence of STC1 from 60 to 88aa among different species. **l** Degradation profile of wild-type STC1 protein and STC1 with T86A mutant (0, 0.5, 1, and 2 h). **m** The effect of STC1 T86A or T86D mutant on its binding to A20. **n** The interaction between STC1 and GSK3β. **o** The co-localization of GSK3β (green) and STC1 (red) (×630) detected by immunofluorescence assay. Blue dye, DAPI indicates the nucleus. **p**–**r** The effect of GSK3β on STC1 protein degradation and expression. **s** The effect of STC1 T86A or T86D mutant on the binding to GSK3β. **t**, **u** The effect of A20 on the interaction between STC1 and GSK3β. **v** The effect of MG132 (20 μM) and chloroquine (40 μM) on STC1 expression. **w** The effect of A20 on ubquitination of STC1 protein in 293T cells. Whole cell lysates were harvested after incubation with MG132 (30 μM for 12 h)

The mRNA level of STC1 were remarkably decreased in A20-KO cells and increased when A20 expression was restored in CRC cells (Fig. 5d). Interestingly, the degradation of STC1 protein was significantly delayed by A20 overexpression (Fig. 7a, b). As the interaction of A20 and STC1 proteins and their co-localization were observed in CRC cells (Fig. 7c, d and supplementary Fig. 4a, b), it is logical to postulate that STC1 protein stability is regulated by its binding to A20. In order to figure out the binding domains between A20 and STC1 proteins, A20 expression vectors encoding different protein domains (Fig. 7e) were constructed and transfected into 293T cells. The binding of A20 and STC1 were diminished in 293T cells transfected with an A20 vector harboring the 1-97aa domain deletion (Fig. 7f, g). Moreover, the upregulation of STC1 expression was disappeared by A20 harboring 1-97aa domain deletion (Fig. 7h). The results indicate the 1-97aa domain was essential for the binding of A20 to STC1.

To identify the exact binding region of STC1 protein for A20, the 293 T cells were co-transfected with A20 and wild-type-STC1 or three STC1 mutants, respectively. A schematic diagram of STC1 protein with different domain deleted is shown in Fig. 7i. The results showed a significant reduction of the binding of A20 with STC1 fragment 3 (deletion of 60~88aa; Fig. 7j). Importantly, the domain of 60~88aa is highly conserved sequences among different species, which encompasses a N62-glycosylation site and a Thr86 phosphorylation site (Fig. 7k).

The importance of Thr86 phosphorylation in the degradation of STC1 protein was further investigated by transfecting a STC1 T86A mutant vector into 293T cells. The degradation of STC1 protein bearing the T86A mutant was significantly retarded (Fig. 7l). However, the interaction between A20 and STC1 was not affected by STC1-T86A or T86D mutant (Fig. 7m).

It has been reported that GSK3β is activated by A20 and it induces target protein phosphorylation, followed by degradation through the ubiquitin ligase SCF–β-TrCP1.^25^ In our CRC cell model, GSK3β was shown to interact with STC1 and decreased STC1 protein level by accelerating its degradation (Fig. 7n–r). Moreover, the interaction between A20 and STC1 was decreased by STC1-T86A or T86D mutant (Fig. 7s). The interaction of STC1 and GSK3β was attenuated by A20 overexpression (Fig. 7t, u). In order to explore the mechanism of STC1 degradation, CRC cells were treated with MG132 at 20 μM or chloroquine at 40 μM, respectively. STC1 expression was notably increased by MG132 treatment which was similar to that of A20 overexpression (Fig. 7v). The ubiquitination of STC1 protein was also significantly inhibited in the presence of A20 overexpression in in vitro ubiquitin assay (Fig. 7w). Therefore, our findings suggest A20 upregulated STC1 expression by inhibiting GSK3β-mediated STC1 Thr86 phosphorylation, and thus retarding the degradation of STC1 protein via ubiquitination.

## Discussion

The majority of CRCs are considered to be immune-quiescent tumors and they are not responding to single checkpoint blockade therapy. In order to achieve clinical benefit of immunotherapy for CRC, it is important to clarify the mechanism of resistance of ICIs and identify suitable biomarker for patient selection.

In our study, PD-1 antibody gave rise to pronounce anticancer effect in CRC cells with A20-deficiency in vitro and in vivo. Immune cells infiltration is a critical determining factor governing the response to PD-1 inhibitor.^26^ A remarkable increase of CD8 ( + ) T cells and macrophages in tumor specimens were observed in A20-silent tumor xenograft-bearing mice and CRC patients with low A20 expression. High A20 expression was demonstrated to impair CD8(+) T cells function and resulted in poor response to PD-1 inhibitor in melanoma patients.^27^ Targeting A20 has been demonstrated to prominently enhance the efficacy of immunotherapy by modulating PD-L1 expression to activate infiltrating CD8(+) T cells.^27^ Microsatellite stability (MSS) CRC is considered an “immune cold” tumor type, which is characterized by low tumor mutation burden and low neo-antigen generation.^9,10^ The immunosuppressive tumor microenvironment in MSS CRC abrogates T cell activity and poses a major barrier to effective immunotherapy. Our study demonstrated that macrophages play a key role in A20-mediated immune evasion. A20 overexpression was associated with poor macrophage infiltration in CRC tissues. The inhibition of tumor growth mediated by A20 deficiency could be restored by the deletion of macrophages with CSF1R antibody in mice. STC1 was reported to impair macrophage to process antigen presentation.^18^ The expression of STC1 and A20 was positively correlated in CRC cell lines and tumor samples of CRC patients. Consistent with this observation, higher STC1 expression was also related to worse prognosis in CRC patients. The sensitivity of CRC cells to PBMCc could be restored by STC1 silencing in the presence of A20. Therefore, STC1 was essential in A20-mediated immune escape. Evasion of immune-surveillance is critical for cancer development and survival. “Eat-me” signals on cell surface of tumors are the key for their recognition by the immune system. STC1 could capture the “eat-me” signal CRT protein inside cell to inhibit its translocation to cell membrane, which was recognized by macrophages to presenting antigens to CD8 + T cells.^18^ In our study, cell membranous CRT expression was notably increased in A20-silent CRC cells, whereas membranous CRT expression was remarkably reduced in A20-overexpressing CRC cells, thus unequivocally demonstrating the critical role played by A20 in regulating “eat-me” signals. It is known that tumor cells can evade immune clearance by up-regulating the expression of immunomodulatory ligands. In CRC, the shift of CRT to the cell membrane and infiltration of lymphocytes are of great importance to immune evasion. In tumor specimens from CRC patients, higher A20 expression was closely associated with lower CD163 (+) macrophages infiltration. More importantly, the tumor growth inhibition could be attenuated by depleting macrophages with CSF1R antibody in mice bearing A20-silent tumor, suggesting that A20 regulated immune response via CRT to affect the antigen presenting process by macrophages. Increased expression of CRT on cell membrane by decreasing STC1 protein level is likely due to suppression of A20-mediated upregulation of STC1. Data from our detailed mechanistic investigation demonstrated that inhibition of A20 facilitated GSK3β-mediated STC1 protein degradation and it was accompanied with increased CRT protein translocation to cell membrane. To this end, GSK3β and A20 were known to be activated by the NF-κB pathway.^28^ GSK3β can phosphorylate and nucleate the substrate protein, which mediates the degradation of the substrate protein by SCF-β-trCP1.^29^ Importantly, we further showed that A20 could bind with STC1 in a competitive manner, thus inhibiting phosphorylation of STC1 at Thr86 by GSK3β and subsequently leading to STC degradation.

In our study, RNA-sequencing was conducted to explore the mechanism of immune evasion mediated by A20. Some genes involved in innate immune response were altered by A20 knockout, such as LCN2, KRT6A, and STC1. LCN2 was reported to induce inflammatory activation and brain metastases in multiple cancer types.^30^ KRT6A was a gene that mediates tumor-associated macrophage activity and tumors with high KRT6A have a hot immunophenotype with increased abundance of immune cells and increased activity of immune-related pathways.^31^ How they work in A20-mediated immune evasion need further study. In addition, the STC1 mRNA levels were also increased by A20 overexpression. A20 may work with other transcription factors to promote STC1 transcription.

The “eat me” signal is an adaptive immune resistance mechanism produced by tumor cells in response to endogenous anti-tumor activity. In fact, several studies have demonstrated the essential role of tumor-derived molecules in the regulation of peripheral immune cells and their anti-tumor immunity.^32^ Therefore, in order to improve the efficacy of immunotherapy, a more thorough understanding about the complex relationships between different sub-type immune cells and tumor cells in the TME is necessary. Restoration of APCs infiltration might be necessary to initiate an immune response.^33^ Due to the negative correlation between A20 expression and tumor-infiltring immune cells, tumoral expression of A20 may be considered as a biomarker to guide appropriate use of anti-PD-1 therapy in treating CRC.

In summary, our findings demonstrated that A20 promotes colorectal cancer immune evasion by upregulating STC1 expression to block membrane translocation of “eat-me” signal, which may represent a novel predictive biomarker for selecting CRC patients who are most likely to benefit from ICI therapy alone or its combination with other anticancer treatment.

## Materials and methods

### Ethics approval and consent to participate

All animal experiments were approved by the Institutional Committee of the Sun Yat-sen University Cancer Center and were in compliance with the protocol approved by the Guangdong Provincial Animal Care and Use Committee and experimental guidelines of the Animal Experimentation Ethics Committee of Sun Yat-sen University Cancer Center (No.L102012019090D). Our study was approved by The Institutional Review Board of Sun Yat-Sen University Cancer Center (No.YB2020-007-01). This study conforms to the Declaration of Helsinki.

### Cell culture

The human colorectal cancer cell lines (CaCO2, LoVo, SW480, SW620, DLD1, HT-29, THC8307, and HCT116) and 293T cells were purchased from ATCC. And all cell lines were validated by short-tandem-repeat (STR) analysis. The CT-26-luc-GFP cells were purchased from Genecopoeia, USA. All cells were cultured in RPMI-1640 medium supplemented with 10% FBS. Peripheral blood mononuclear cells (PBMCs) were maintained with 100 IU/ml human IL-2. All cells were cultured in a 37 °C humidified incubator containing 5% CO_2_.

### Chemicals and reagents

The PD-1 inhibitor (pembrolizumab) was purchased from Selleckchem (Houston, TX, USA). The human anti-TNFAIP3, anti-HA-tag and anti-GSK3β antibodies were bought from Cell Signal Technology (Danvers, MA, USA). The antibody against GAPDH, CRT, and Flag-tag were purchased from Proteintech (Chicago, IL, USA). The human STC1, CD163 and anti-mouse CD3, CD4, CD8, F4/80, and granzyme B antibodies were purchased from Abcam (Cambridge, UK). The anti-human CD3, CD4 and CD8 antibodies were purchased from Zsbio (Beijing, China). The fetal bovine serum (FBS) was obtained from Gibco BRL (Gaithersburg, MD, USA). The RPMI-1640, penicillin, streptomycin and trypsin were obtained from Cornning (Corning, NY, USA). The anti-mouse PD-1 antibody, anti-mouse CSF1R (CD115) antibody and rat IgG2a isotype control were obtained from BioXcell (Lebanon, NH, USA).

### Western blotting

Briefly, RIPPA lysis buffer was used to collect whole cell lysates and protein concentration was determined with Pierce BCA Protein Assay Kit (Thermo Fisher Scientific, Waltham, MA, USA). Proteins were separated on 10% gel by SDS-Polyacrylamide electrophoresis and transferred to PVDF membrane. After blocking with 5% skim milk for 1 h, the cell membrane was detected with specific primary antibody and HRP binding secondary antibody. All primary antibodies were diluted at 1:1000 and secondary antibodies were diluted at 1:5000. The final analysis was carried out by three independent experiments. The ECL detection kit was used to visualize protein bands.

### Immunofluorescence

CRC cells were plated in confocal dishes and then incubated with Mito-tracker (100 nM) (Thermofisher, Waltham, MA, USA) for 30 min at 37 °C. The cells were fixed with 4% paraformaldehyde for 15 min as previously described,^34^ then washed twice with PBS and permeabilized with 0.2% Triton X-100 in PBS for 15 min at room temperature. Blocking with 5% bovine serum albumin at room temperature for 45 min and incubation with primary antibody at 4 °C overnight. Follow washing for 2 times, the dishes were incubated with Alexa Flour 647 or 488‐labeled secondary antibody, respectively for 1 h. DAPI staining (1 ug/mL,10 min) was performed after two washes and observed on a Zeiss laser scanning confocal microscope (LSM880, Germany) or an ultra-high resolution microscope (Nikon N-SIM).

### Transwell assay

Transwell assay was used to detect the migration and invasion of CRC cells as previously described.^35^ CRC cells (migration: 6 × 10^4^ cells, invasion: 1 × 10^5^ cells) in 200 μL of FBS-free medium were seeded into a 24-well transwell cell culture chamber (8 μm pore size, BD), and then 650 μL of 20% FBS medium was added to the lower chamber. The insert chamber was coated with 0.5% Matrigel (657,638, Greiner Bio-One, UK) for invasion assay. Cells at the bottom of the chamber were fixed with 4% paraformaldehyde and stained with 0.5% crystal violet. At least five random fields of view were taken under the microscope and cells were counted. All experiments were repeated three times.

### Immunohistochemistry and scoring

The tumor specimens were fixed with 10% formalin buffer overnight and paraffin-embedded and cut into 4 µm sections. Then the sections were deparaffinized twice with xylene and rehydrated in gradient ethanol. High pressure-mediated antigen retrieval in EDTA Antigen Retrieval Solution (pH 9.0) (Zsbio, China) was performed. Then 3% hydrogen peroxide was used and blocked with serum-free protein block (DAKO). After incubation with the primary antibodies at appropriate dilution (A20, 1:100, STC1, 1:400, CD163, 1:400, CD3, 1:100, CD8, 1:200, CD4, 1:100, F4/80, 1:100) for 50 min at 37 °C, and secondary antibodies for 30 min at room temperature, the sections were stained with DAB (Zsbio, China). All specimens were scored double-blind by two experienced pathologists. The staining was firstly assessed as a whole at low microscopic magnification (×100), then 5 random fields at higher magnification (×200 or ×400) were averaged. The interpretation of immunoreactivity was based on an H-score (histochemical scoring assessment), which incorporates both staining intensity and the percentage of stained cells at each intensity level. The staining of intensity was 0 (absence), 1 (low or weak expression), 2 (moderate expression), or 3 (strong expression). The percentage of stained cells was 1(≤25%), 2(≤50%), 3 (≤75%), or 4 (>75%). Absolute cell counts were recorded for CD3, CD4, CD8, granzyme B, CD163, and F4/80 positive cells.^36^

### Tumor cells and PBMCs co-culture system

Fresh PBMCs were obtained from healthy donors by Ficol-Paque Plus reagent (Solarbio, China). The tumor cells and PBMCs co-culture system was established as previously reported^37^ The Anti-CD3 (OKT3) 5 µg/mL (Biolegend, San Diego, CA, USA) was pre-plate-bound for 4 h and then PBMCs were plated at 1 × 10^5^ cells/well The target cells (CRC cells) were incubated with the effector cells (PBMCs) at a ratio from 1:1 to 4:1 in 12-well plates for 3–4 days in technical triplicates. Human PD-1 inhibitor (pembrolizumab) (100 μg/mL) was administrated into the co-culture medium. After incubation for 3–4 days, PBMCs were discarded and the rest of the tumor cells were stained with gimesa. The relative intensity of the staining was analyzed by Image J software.

### Animal studies

All animal studies were used the ARRIVE1reporting guidelines.^38^ Female immunocompetent BALB/C mice T (5-6 week-old) were purchased from the Laboratory Animal Unit of the Guangdong Province (China). For the metastatic mouse model, CT26-luc-GFP cells infected with shRNA control or A20 shRNA (1 × 10^5^ cells per mouse) were resuspended in 100 µL PBS and injected intravenously into the tail vein of mice. The rat anti-IgG isotype control or anti-PD1 antibody were injected intravenously into mice bearing CT26-luciferase-GFP cells every 4 days after tumor cell injection (10 mg/kg).^39^ Bioluminescence imaging was obtained by IVIS-Xenogen 100 system (Caliper Lifesciences, Hopkinton, MA, USA).^40^ To plot the survival curve, the death criteria were defined as a near-death state. The mice were euthanized when they presented with changes in physiological and behavioral characteristics including stooping posture, reduced activity, increased facial swelling, ear rearward position, abdominal swelling, squinting, breathing difficulties, etc.^41^

For the subcutaneous tumor xenograft model, female BALB/C or NSG mice were subcutaneously injected with CT26 cells transfected with shRNA control or A20 shRNA (3 × 10^5^ cells/mouse) into the flank as previously described.^42^ Female NSG mice (5–6 week-old) were obtained from the Department of Experimental Animal Center, First Affiliated Hospital of Sun Yat-sen University. After 7 days, the tumor size was measured every 2-3 days and calculated as 0.5 × length × width^2^. Mice were sacrificed when tumor volumes >2000 mm^3^. To deplete macrophages, mice were intraperitoneally injected with 800 μg monoclonal CSF1R antibodies on day −4 and 400 ug on day 3, 10, 17, 24 relative to tumor inoculation.^18^

### TCGA data

The TCGA colorectal carcinoma data sets referenced in this study can be found in the public database of the TCGA portal (https://portal.gdc.cancer.gov/). RNA sequencing data (GSE206390) have been uploaded to the National Center for Biotechnology Information Gene Expression Omnibus (NCBI–GEO) database (http://www.ncbi.nlm.nih.gov/geo/) .

### RNA sequencing and data analysis

The RNA-sequencing was performed on HCT116-wild type (WT), HCT116-A20 knockout (KO) and HCT116-A20-KO-rescue (RE) cells by the Novogene Facility (Beijing, China). Each cell samples had a purity of >90%. After assessing RNA integrity with the RNA Nano 6000 assay kit of the Bioanalyzer 2100 system (Agilent Technologies, Santa Clara, CA, USA), the mRNA was purified using poly-T oligomeric attachment magnetic beads. Each library needs to purify mRNA from 1000 ng of total RNA. Each poly-A-enriched sample was fragmented, synthesized into double-stranded cDNA, incubated with Klenow DNA polymerase and DNA fragments, ligated to an Illumina adaptor, and purified with paramagnetic beads. Clustering of index-coding samples was performed on the cBot Cluster Generation System using TruSeq PE Cluster Kit v3-cBot-HS (Illumia). After clustering generation, library preparations were sequenced on the Illumina Novaseq platform, and 150 bp paired-end reads were generated. Reads mapped to each gene were calculated with Feature Counts vl.5.0-p3. FPKM for each gene and the count of reads mapped to that gene were then calculated based on the length of the gene. Differential expression analysis was performed using the DESeq2 R package (1.20.0). The final *p*-value is adjusted by Benjamin and Hochberg’s method to control the false discovery rate. Genes with an adjusted *p*-value < 0.05 and absolute fold change > 2 were set as the threshold for significantly differential expression. RNAseq data analysis was performed using R3.3.1 and Bioconductor 2.32.0. The Venn Diagram Package was used to generate Venn diagrams.

### Quantitative real-time PCR

The quantitative real-time PCR analysis was performed as previously described.^43^ The total cellular RNA was extracted and reverse-transcribed into cDNA, then real-time quantitative PCR was performed using ChamQ SYBR qPCR Master Mix (without ROX) (Vazyme). Data were analyzed using the 2-ΔΔCT method after standardization with β-actin expression levels in each sample. The primers were listed in Supplementary Table 1.

### Flow cytometry

CRC cells and PBMCs were collected and suspended in PBS. All cells were incubated with anti-human CRT (Biolegend, San Diego, CA, USA) or anti-human HLA-A2 (Biolegend, USA) for 30 min. The single-cell suspensions of mouse spleen were processed by mashing the spleen through 40 μm cell strainers and then incubated in RBC lysis buffer (Sigma-Aldrich, St Louis, MO, USA). Samples were detected on a CytoFLEX cytometer (Beckman Coulter, Germany). The antibodies: CD8 (clone 53-6.7), CD3 (clone 3C7), CD4 (clone IM7) were obtained from Biolegend (USA). The Flowjo software (Treestar, Woodburn, OR, USA) was used to analyze flow cytometry data.

### Plasmid construction and viral infection

The human A20 overexpression vector (pCMV-3xFlag-A20) was purchased from GeneCopoeia Inc (Guangzhou, China). Single-guide RNA (sgRNA) targeting A20 gene were cloned into the LentiCRISPR v2 lentiviral backbone vector (Addgene, Watertown, MA, USA). The shRNAs of A20 or STC1 and a negative shRNA control (shctrl) were cloned into pSIH-H1-puro lentivector (Addgene, USA). The sgRNA and shRNA sequences were listed in Supplementary table 2. The 293T cells were transfected with lentivirus vector, packaging (psPAX2.2), and envelope (pMD2.G) plasmids using Lipofectamine 3000 Transfection Reagent (Life Technologies, Carlsbad, CA, USA). The lentivirus was collected after 48 and 72 h by filtration with a 0.45-μm Steriflip Filter (Millipore, Burlington, MA, USA). Then CRC cells were cultured with the medium containing lentivirus and 1 ng/mL polybrene (Sigma, St.Louis, MO, USA) at 37 °C. After 24 h, cells were screened by puromycin.

### ELISA

The secretion of IFN-γ, TNF-α, IL-6, and granzyme were analyzed by ELISA kit (Biolegend, San Diego, CA, USA) using supernatants from the co-culture system of cancer cells and PBMCs. Supernatants were harvested after 48 h and diluted (1:5) prior to analysis and used at 100 μL per well of a 96-well plate. Following 3 washes, detection antibody (50 µL) was added and incubated for 1 h at room temperature. Then washed the plate 3 times and added 100 µL avidin horseradish peroxidase (HRP) for 30 min. After 3 washes, 100 µL tetramethylbenzidine (TMB) substrate solutions was added and incubated at room temperature for 15 min. Add 100 µL stop solution and read the plates at 450 and 630 nm on a Bio-Rad plate reader (Bio-Rad, Hercules, CA USA).

### Statistical analysis

The data were calculated with IBM SPSS Statistics 19 software by Student’s t-test or one-way ANOVA analysis and presented as mean ± SD. The Kaplan–Meier method with log-rank analysis was used to estimate overall survival. The relationship between A20 and STC1 was conducted using Pearson correlation coefficient. Experiments were repeated in triplicate. Statistical significance was defined as *p* < 0.05.

### Supplementary information

supplementary materials

## Data Availability

The RNA-seq data are uploaded onto Gene Expression Omnibus (GEO) at GSE206390 (http://www.ncbi.nlm.nih.gov/geo/). All the original data were deposited in the database RDDB2023800132 (http://www.researchdata.org.cn/). And all original and uncropped films of Western blots were presented at supplementary Figs. 5–7.
