# Supplementary material for: A20 promotes colorectal cancer immune evasion by upregulating STC1 expression to block “eat-me” signal
Source: Signal Transduct Target Ther. 2023 Aug 23;8:312. doi: 10.1038/s41392-023-01545-x (PMC10444827; doi:10.1038/s41392-023-01545-x)
Supplement: Supplementary file 1 — supplementary materials [file 41392_2023_1545_MOESM1_ESM.doc]

Supplementary Materials for

**A20 promotes colorectal cancer immune evasion by upregulating STC1 expression to block membrane translocation of “eat-me” signal**

Min Luo^1^, Xueping Wang^1^, Shaocong Wu^1^, Chuan Yang^1^, Qiao Su^2^, Lamei Huang^1^, Kai Fu^1^, Sainan An^1^, Fachao Xie^1^, Kenneth Kin Wah To ^3^, Fang Wang^1，^*, Liwu Fu^1^,*

Correspondence to: [wangf@sysucc.org.cn](mailto:wangf@sysucc.org.cn) or [Fulw@mail.sysu.edu.cn](mailto:Fulw@mail.sysu.edu.cn).

**This PDF file includes:**

Figures. S1 to S7

Table S1 to S2


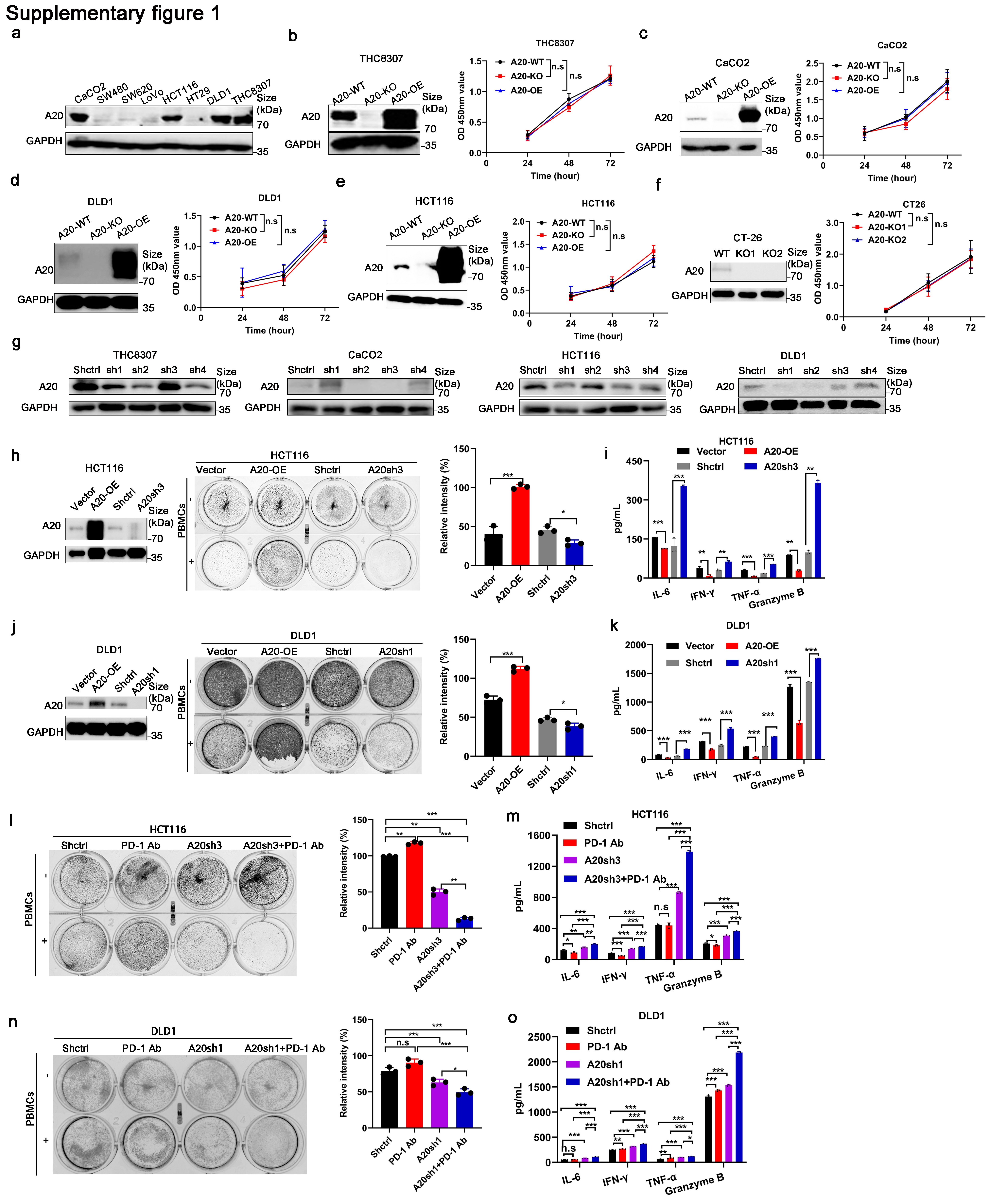


**Figure. S1.**

**A20 inhibited anti-tumor immune response *in vitro*.**

(a)A20 expression in different CRC cells detected by Western blotting. (b) -(f) The effect of A20 on CRC cell proliferation. n=5. (g) Western blotting assay of CRC cells infected with scramble or shRNAs targeting A20. (h) -(k) The effect of A20 expression on lymphocytoxicity and cytokine release from the co-culture medium of HCT116 or DLD1 cells and PBMCs. (l) - (o) The effect of PD-1 inhibitor on lymphocytoxicity and cytokine release from the co-culture medium of A20-knockdown CRC cells and PBMCs. All experiments were performed in triplicate. Data was represented as the mean ± SD. *p< 0.05; **p< 0.01; ***p< 0.001; n.s, not significant.


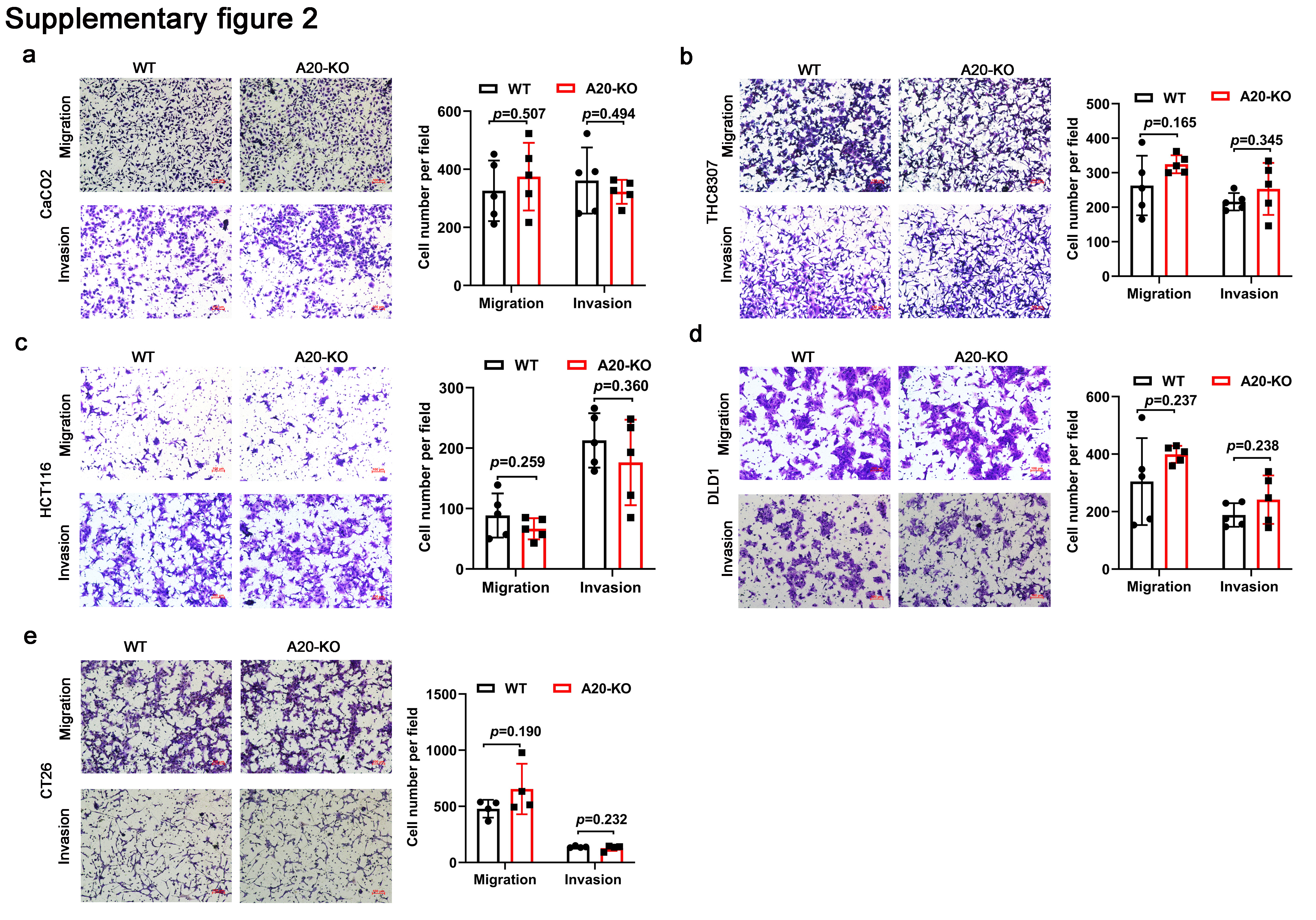


**Figure. S2.**

**A20 doesn’t affect cell migration and invasion ability of CRC cells.**

(a) - (e) CRC cells migration and invasion ability, n=5 or 4. All experiments were performed in triplicate. Data was represented as the mean ± SD. *p< 0.05; **p< 0.01; ***p< 0.001; n.s, not significant.


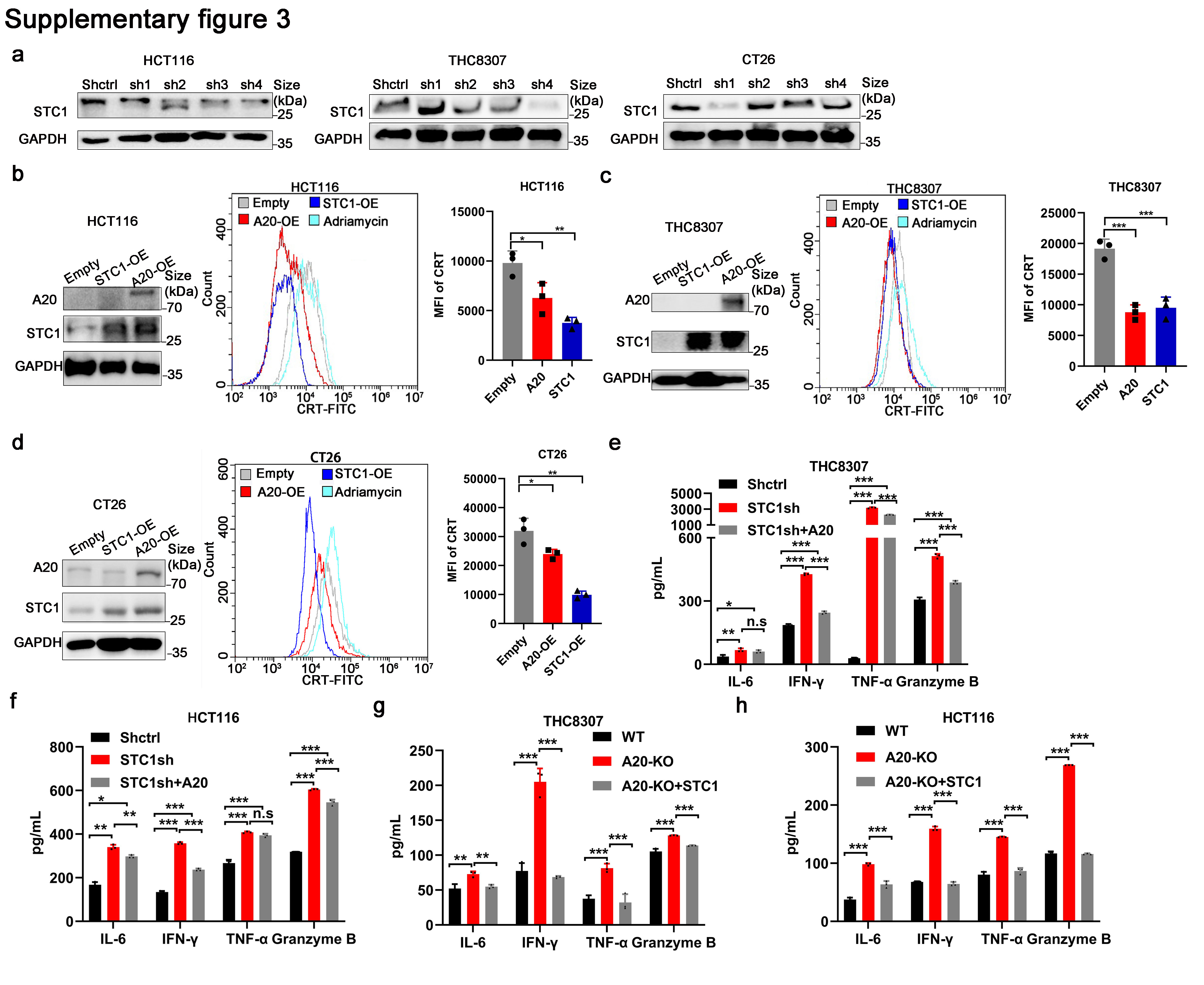


**Figure. S3.**

**A20 inhibited anti-tumor immune response via STC1.**

(a) Western blotting assay of CRC cells infected with scramble or shRNAs targeting STC1. (b) - (d) The effect of STC1 or A20 overexpression on the translocation of CRT to cell membrane detected by flow cytometry. Adriamycin was used as a positive control. (e) -(h) The ELISA analysis of T cell activation related cytokines from the co-culture medium of CRC cells and PBMCs, n=3. All experiments were performed in triplicate. Data was represented as the mean ± SD. *p< 0.05; **p< 0.01; ***p< 0.001; n.s, not significant.


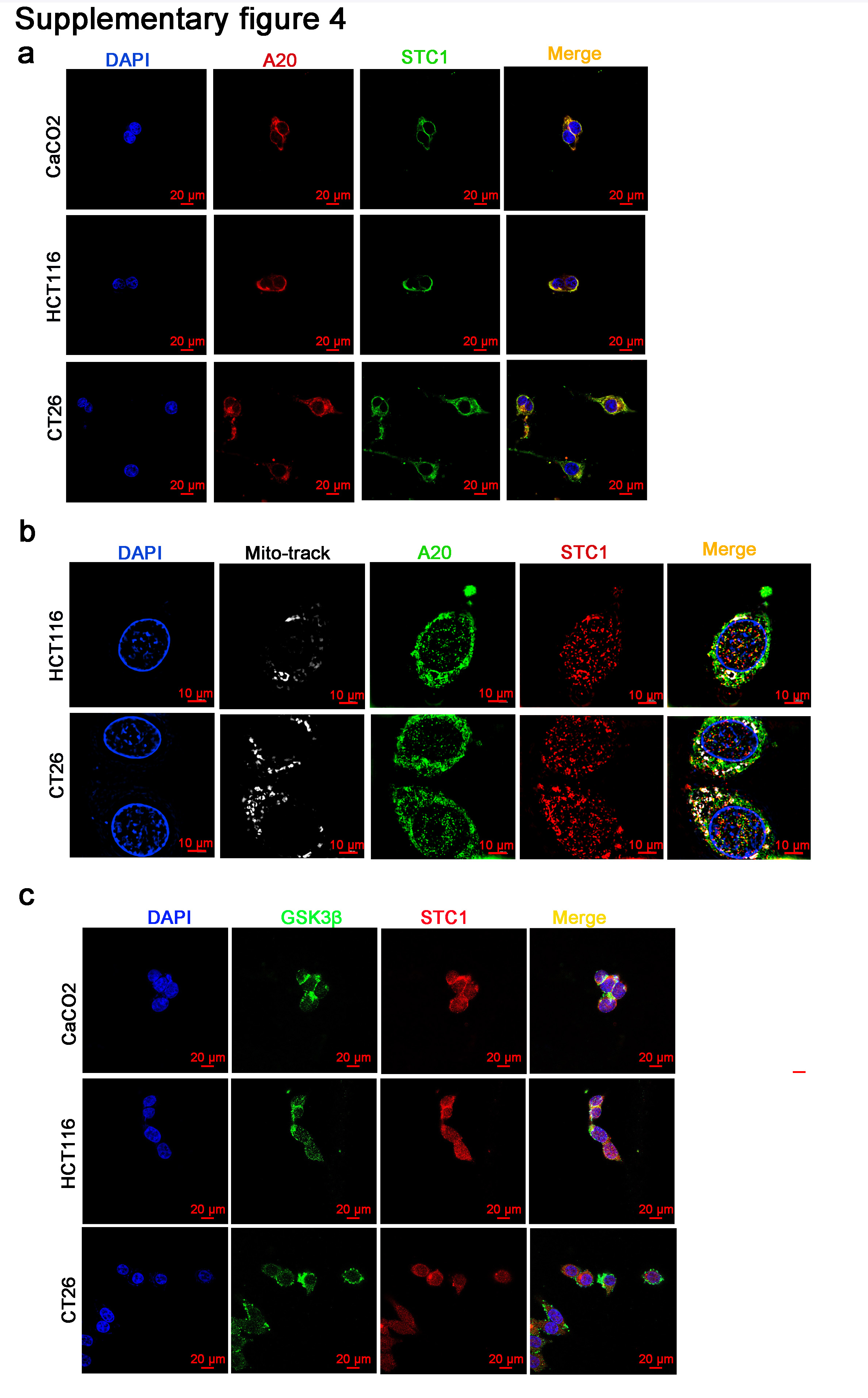


**Figure. S4.**

**The interactions of STC1 and A20 or GSK3β protein in CRC cells .**

(a)(×630) and (b)(×1000) The co-localization of STC1 and A20 detected by immunofluorescence assay. (c) The co-localization of STC1 and GSK3β (×630). Blue dye, DAPI indicates the nucleus, MitoTracker (white).

**
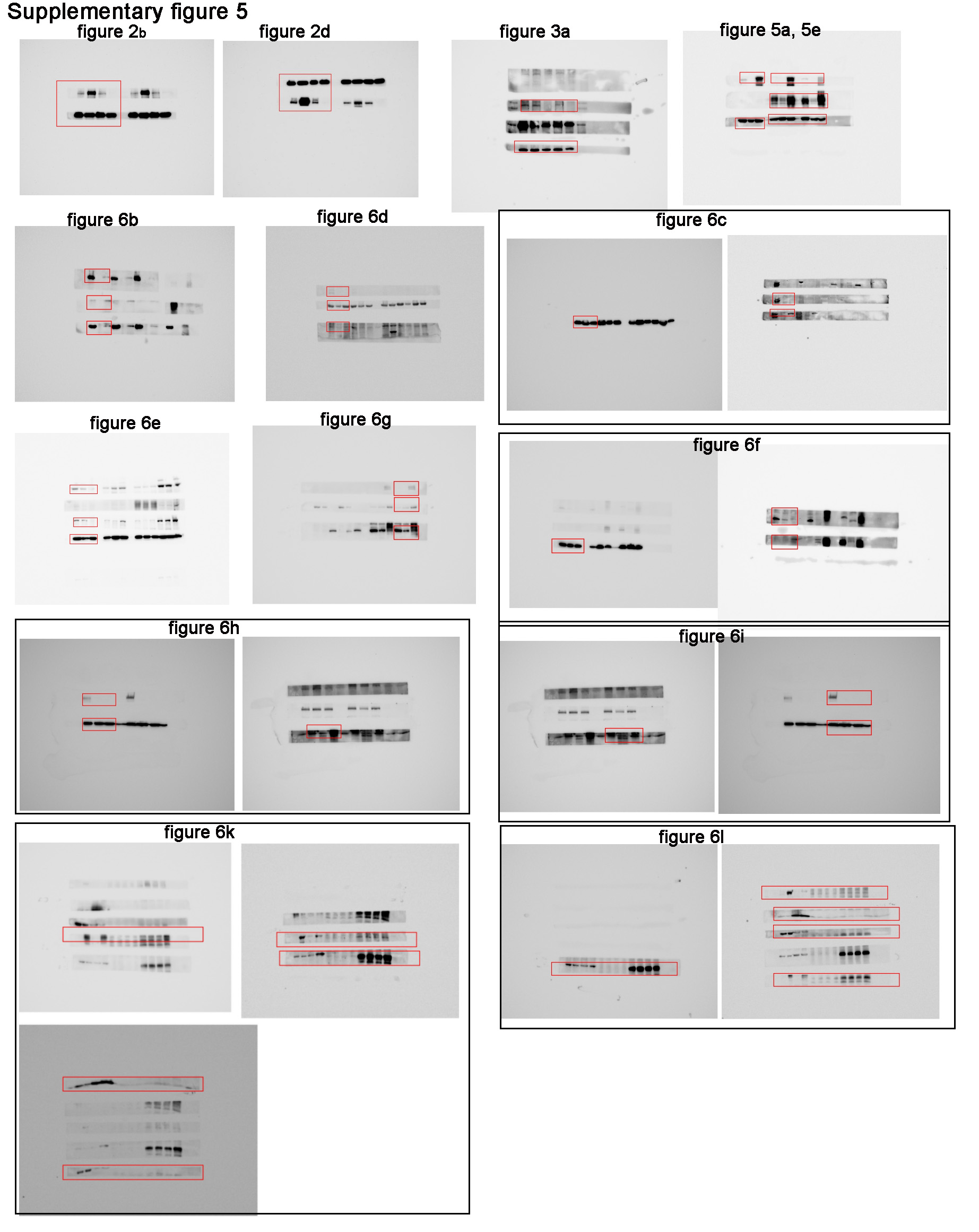
**

**Figure. S5.**

**The original and uncropped films of Western blots of figure 2 to 6.**

**
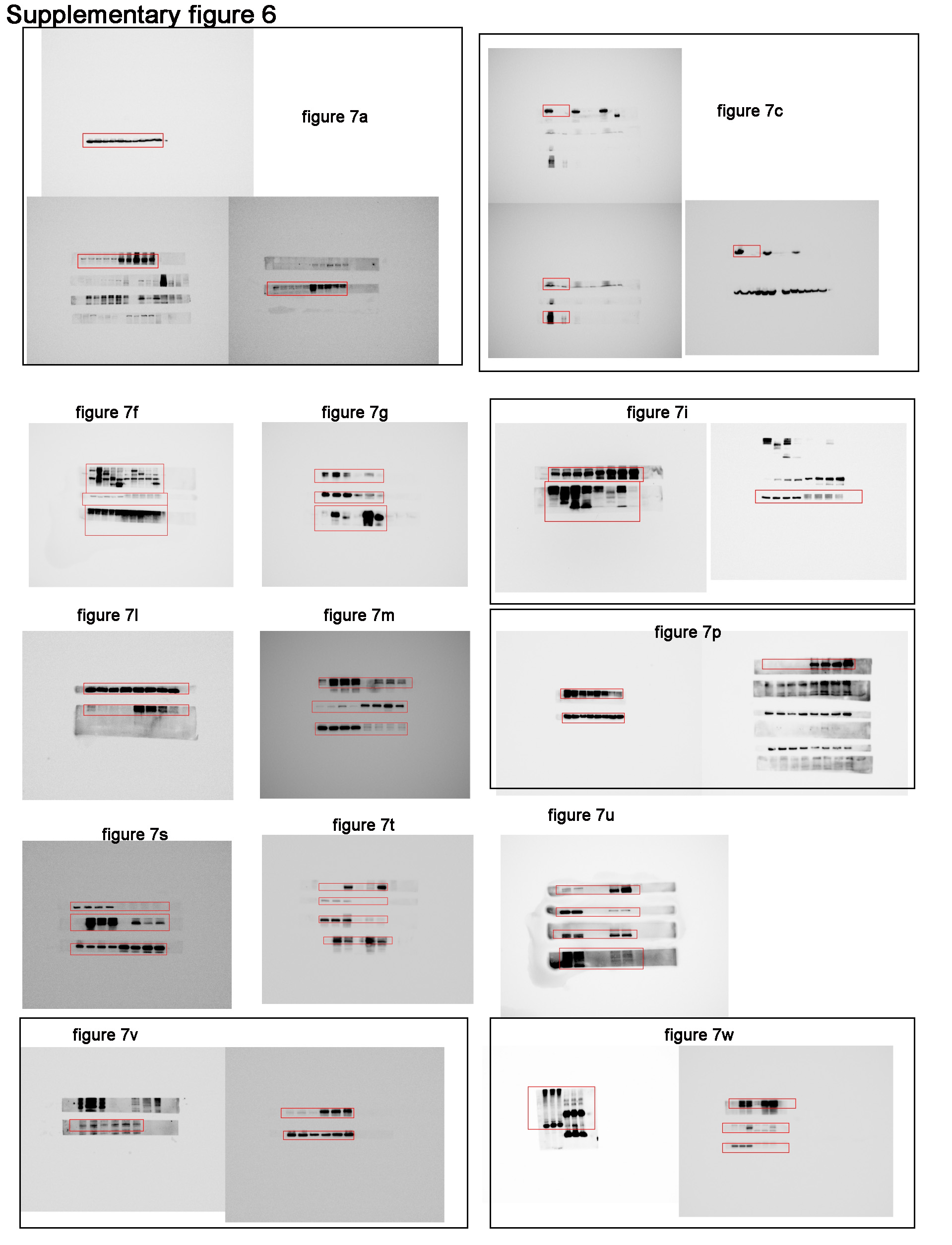
**

**Figure. S6.**

**The original and uncropped films of Western blots of figure 7.**

**
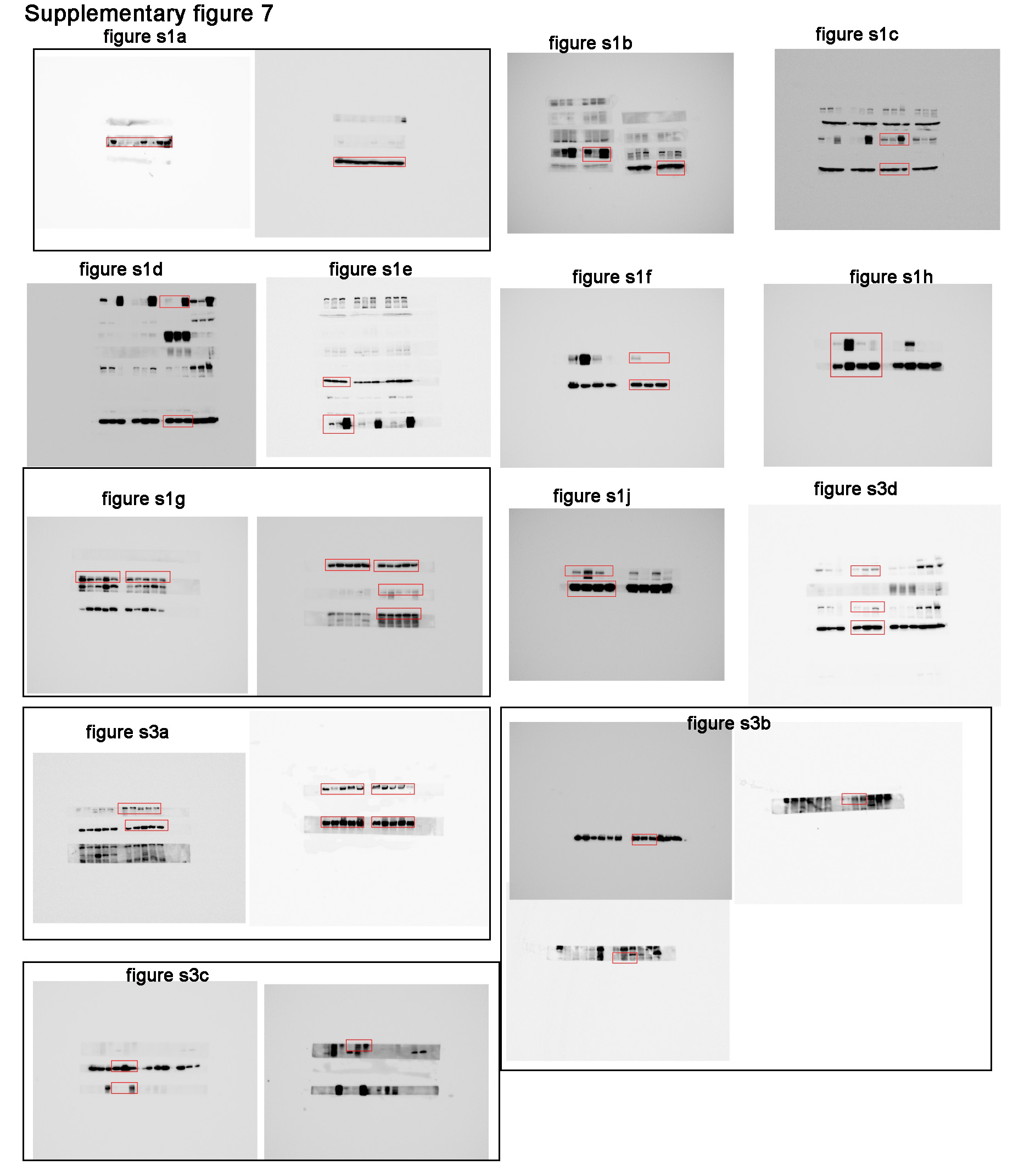
**

**Figure. S7.**

**The original and uncropped films of Western blots of supplementary figure 1 to 3.**

**Table S1 The primers for quantitative PCR**

| **Genes** | **Forward** | **Reverse** |
| --- | --- | --- |
| A20 | GATAGAAATCCCCGTCCAAGG | CTGCCATTTCTTGTACTCATGC |
| β-actin | TCCTGTGGCATCCACGAAACT | GAAGCATTTGCGGTGGACGA |
| STC1 | CTGAAGTGGTTCGTTGCCTC | CTGAGTGTCAAATTTAGCAGCG |
| LCN2 | ACTCGCCACCTCCTCTTC | TTGGTTGTCCTGGAAGTTCT |
| HNF1B | GCCTCAACACCTCCCAAG, | TGTGTGAGTTCTGCAGCTGA |
| STXBP5L | ACAGTTCGGCATGGTTTTC | ATTCGTATAGCACCTGTTCTCG |
| CAVIN2 | CTGACATGCGGCAGGAAA | TCTTGTGCTGGTTCTCCTGC |
| MYO7B | AGTCATTTCCTCTTGGAGCC | CTTGTCAGGATGGGGGTCT |
| NECAB1 | GACTACCAAGAAGCCTCCAATT | CTTTCTTGACGGGTTTGCTC |
| CHRNA9 | AATGCAAGCCTGAGCTCTCC | CTCTCAGTCTGGAAGCAGCAAA |
| MSR1 | ATCCTAAAAACAGCCCTTCC | CTGCCACTATTCCAATGAGAG |
| KRT6A | GATGTGGATGCTGCCTACATG | TGCATCATACAAGGCTCTCAG |

Table S2. sgRNAs and shRNAs sequences

| **Genes** | **sgRNA or shRNA** |
| --- | --- |
| Human A20 sgRNA1 | GGGACTTTGCGAAAGGATCG |
| Human A20 sgRNA1 | GAGGCAATTGCCGTCACCTG |
| Mouse A20 sgRNA1 | GGCTGAACAACTTCTTCCTC |
| Mouse A20 sgRNA2 | GAGCTTCTTCTGGCTTTCCA) |
| ShRNA control | CAACAAGATGAAGAGCACCAA |
| Human A20 shRNA1 | GGTTGTAGATGCTGGTCTTTG |
| Human A20 shRNA2 | GGTGGAAACTTCTTTCCTTGT |
| Human A20 shRNA3 | GCAGCTGAGCAATGACCATAT |
| Human A20 shRNA4 | GCTGAACTCCAAGGGCTATAC |
| Human STC1 shRNA1 | GAGGTGCTCCACTTTCCAAAG |
| Human STC1 shRNA2 | GCTACAGCAAGCTGAATGTGT |
| Human STC1 shRNA3 | GCCCAATCACTTCTCCAACAG |
| Human STC1 shRNA4 | GCACAATCAGAGACAGCCTGA |
| Mouse A20 shRNA1 | ATGGGATCATCTATCACTTTA |
| Mouse A20 shRNA2 | AGCTATCACTCATGGATATAA |
| Mouse A20 shRNA3 | TGCCACGCCCAGCAGATTAAT |
| Mouse A20 shRNA4 | GCACTCTATGTTTCATCGAAT |
| Mouse STC1 shRNA1 | GGTTTCCTGCCAGCAGGTATA |
| Mouse STC1 shRNA2 | GGTATATGGGAGCAGTGTTAA |
| Mouse STC1 shRNA3 | GCAGTGTTAAAGGCAGCCTAA |
| Mouse STC1 shRNA4 | GCCTAACGGAATGGGCTTTAC |
